# Estimates of Cancer Incidence in Ethiopia in 2015 Using Population-Based Registry Data

**DOI:** 10.1200/JGO.17.00175

**Published:** 2018-03-28

**Authors:** Solomon Tessema Memirie, Mahlet Kifle Habtemariam, Mathewos Asefa, Biniyam Tefera Deressa, Getamesay Abayneh, Biniam Tsegaye, Mihiret Woldetinsae Abraha, Girma Ababi, Ahmedin Jemal, Timothy R. Rebbeck, Stéphane Verguet

**Affiliations:** **Solomon Tessema Memirie**, **Timothy R. Rebbeck**, and **Stéphane Verguet**, Harvard T.H. Chan School of Public Health; **Timothy R. Rebbeck**, Dana-Farber Cancer Institute, Boston, MA; **Mahlet Kifle Habtemariam**, Federal Ministry of Health; **Mathewos Asefa**, Tikur Anbessa Specialized Hospital, Addis Ababa University, Addis Ababa; **Biniyam Tefera Deressa**, Gondar University, Gondar; **Getamesay Abayneh**, Haromaya University, Dire Dawa; **Biniam Tsegaye**, Ayder Comprehensive Specialized Hospital, Mekele University, Mekele; **Mihiret Woldetinsae Abraha**, Harar General Hospital, Harar; **Girma Ababi**, Hawassa University, Hawassa, Ethiopia; and **Ahmedin Jemal**, American Cancer Society, Atlanta, GA.

## Abstract

**Purpose:**

Noncommunicable diseases, prominently cancer, have become the second leading cause of death in the adult population of Ethiopia. A population-based cancer registry has been used in Addis Ababa (the capital city) since 2011. Availability of up-to-date estimates on cancer incidence is important in guiding the national cancer control program in Ethiopia.

**Methods:**

We obtained primary data on 8,539 patients from the Addis Ababa population-based cancer registry and supplemented by data on 1,648 cancer cases collected from six Ethiopian regions. We estimated the number of the commonest forms of cancer diagnosed among males and females in Ethiopia and computed crude and age-standardized incidence rates.

**Results:**

For 2015 in Ethiopia, we estimated that 21,563 (95% CI, 17,416 to 25,660) and 42,722 (95% CI, 37,412 to 48,040) incident cancer cases were diagnosed in males and females, respectively. The most common adult cancers were: cancers of the breast and cervix, colorectal cancer, non-Hodgkin lymphoma, leukemia, and cancers of the prostate, thyroid, lung, stomach, and liver. Leukemia was the leading cancer diagnosis in the pediatric age group (age 0 to 14 years). Breast cancer was by far the commonest cancer, constituting 33% of the cancers in women and 23% of all cancers identified from the Addis Ababa cancer registry. It was also the commonest cancer in four of the six Ethiopian regions included in the analysis. Colorectal cancer and non-Hodgkin lymphoma were the commonest malignancies in men.

**Conclusion:**

Cancer, and more prominently breast cancer, poses a substantial public health threat in Ethiopia. The fight against cancer calls for expansion of population-based registry sites to improve quantifying the cancer burden in Ethiopia and requires both increased investment and application of existing cancer control knowledge across all segments of the Ethiopian population.

## INTRODUCTION

Cancer incidence has increased in most countries worldwide, owing to a growing and aging population and to an expansion of key risk factors, such as smoking, obesity, and unhealthy diet.^1,2^ Cancer has become the second leading cause of death, behind cardiovascular disease, with more than 8.7 million attributable deaths worldwide in 2015.^1^ Low-income countries (LICs) contribute up to 60% of this death toll.^1^ This is an alarming prospect, especially for LICs, where the weak health systems are severely resource constrained and already overwhelmed by the large burden of communicable diseases.

Similar to other LICs, the burden of noncommunicable diseases (NCDs) is increasing in Ethiopia. Cancer has become the second leading cause of death in the adult population.^3,4^ NCDs, including cancer, were among health targets of the Sustainable Development Goals^5^; the Global Burden of Disease Cancer Collaboration and Breast Health Global Initiative stated that cancer control strategies were to be prioritized based on local needs and that data on cancer epidemiology would be necessary for the development of national NCD and cancer action plans.^1,6,7^

Local data on cancer epidemiology in Ethiopia are lacking. Studies from the Global Burden of Disease Cancer Collaboration and the Cancer Incidence in Five Continents Collaboration have estimated cancer incidence by cause for countries globally, and both studies used evidence from neighboring African countries to estimate cancer incidence in Ethiopia.^1,8^ A population-based cancer registry system has been used in Addis Ababa (Ethiopia’s capital city) since 2011, and the Global Burden of Cancer Study (GLOBOCAN) estimated the incidence and mortality from major types of cancer for Ethiopia using such available data for 2012.^9^ Over the years, the quality of the registry data has improved through additional staff trainings and ongoing technical support from global experts.^10,11^

The fast-growing and aging population, the surge in the prevalence of risk factors such as obesity and physical inactivity, and the changes in reproductive patterns related to urbanization could increase the cancer burden in Ethiopia.^12^ Recognizing this threat, the government has recently launched a national cancer control plan.^13^ The plan set ambitious objectives to expand a range of preventive interventions, screening tests for early detection, and diagnosis and treatment, with provision of chemotherapy, surgery, radiotherapy, and palliative care. Intervention selection depends on several factors, including disease burden, cost, and effectiveness. Therefore, availability of timely local epidemiologic data is indispensable to prioritize scale-up of interventions in Ethiopia.^14^ To support this process, we have used cancer registry data to estimate the crude and age-standardized incidence rates of the commonest types of cancer in Ethiopia for the year 2015.

## METHODS

### Study Area and Population

Ethiopia has an estimated population of > 99 million inhabitants, and most (84%) live in rural areas.^15,16^ The study was undertaken in Addis Ababa, using a population-based cancer registry that is housed in Tikur Anbessa Specialized Hospital (TASH), the country’s largest tertiary hospital, as well as in university hospitals located in the six major regions of Ethiopia (home to 90% of the country population): Oromia, Amhara, the Southern Nations Nationalities and People (SNNP), Harari, Tigray, and Dire Dawa.^16^ The basis of selection was availability of cancer care services in the regional health facilities.

### Addis Ababa Cancer Registry

Addis Ababa Cancer Registry (AACR) collects data on the population of Addis Ababa City Administration. The population is entirely urban, but with diverse ethnic and religious composition.^16^ In Addis Ababa, cancer care is provided in three public hospitals, including TASH and twelve private facilities. In most cases, a cancer diagnosis was confirmed through pathologic examination of a biopsy specimen, whereas in others it was through clinical, laboratory and imaging modalities. TASH provides the bulk of cancer care, both for Addis Ababa residents and nationally. Albeit provision of care in public hospitals and private facilities is irrespective of geographic location, the AACR unit collects data on patients with cancer only for residents of the city. In the computation of incidence, only patients with cancer from Addis Ababa were included.

The AACR unit has three full-time employees and has trained 20 contact persons in each health institution delivering cancer diagnostic/treatment services. The contact persons collect data on patients with cancer (for all ages) daily from inpatient and outpatient departments, including pathology units, using a standardized format developed by the International Agency for Research on Cancer.^8^ Cases identified in each unit are reported weekly to the AACR unit. AACR staff regularly supervises the contact persons to make sure all cases are registered and to verify data quality. When the contact persons leave the facility, AACR staff provides training to a substitute contact person. Duplicate entries are identified and corrected based on demographic data and patient phone number. On the basis of the topography (site of origin of a cancer) and tumor morphology (type of cancerous cell), each case was coded into a coding schema from the full International Classification of Diseases for Oncology, 3rd edition (ICD-O-3).^8^ Once a data set had been converted into ICD-O-3, it was checked for consistency by code verification (sex and ICD-O-3 topography and morphology) and consistency between items (age *v* incidence dates, sex *v* site, sex *v* histology, age *v* site, site *v* histology).^8,17^ The registry uses CanReg5 system for data entry, analysis, quality control, and management.^17^

### Completeness and Data Quality

In assessing cancer incidence, completeness of registration (the extent to which all incident cases in the population are included in the registry) is a vital factor.^18^ We assessed completeness using the following indices: stability of incidence rates over time, proportion of cases microscopically verified, and proportion of unknown basis of diagnosis. We could not use mortality-to-incidence ratios, because accurate data on cancer mortality were lacking.

### Crude and Age-Standardized Incidence Rates

We calculated incidence of a specific cancer by 5-year age category and sex. The crude incidence rate (CIR) is the rate at which new cases occur in a population during a specific period. CIR is classically expressed as the average number of cases occurring per 100,000 population. It relates to each population as a whole and is influenced by the age structure of each population.

The age-standardized incidence rate (ASIR) is a summary of the individual age-specific rates using an external population called a standard population. This is the incidence that would be observed if the population had the age structure of the standard population and corresponds to the CIR in the standard population. Similar to CIR, ASIR is expressed as the number of new cases per 100,000 population (details are provided in the Data Supplement). We used the 1960 world population for age standardization.^19^ Data on the population distribution of Ethiopia and Addis Ababa were obtained from the United Nations Population Division and the Central Statistics Agency of Ethiopia.^15,20^ We estimated, nationally, the expected number of cases and the corresponding 95% CIs on the basis of the average incidence for each specific cancer over the 4-year period 2012 to 2015 from AACR data. Using data available for breast cancer cases only, we also assessed stage of the disease at the initial presentation.

### Regional Data

To supplement the data from AACR, we collected additional hospital-based data from pathology units of five regions (Oromia, Tigray, Amhara, SNNP, Harari) and Dire Dawa for the year 2015, which allowed comparison and assessment of variations in the commonest forms of cancer across regions. We used STATA 13 (STATACorp, College Station, TX) and Microsoft Office Excel 2007 for further data processing and analysis. Ethical clearance was obtained from the scientific and ethical committee of the Ethiopian Public Health Institute and from the Office of Human Research Administration, Harvard T.H. Chan School of Public Health (Protocol # IRB16-1883).

## RESULTS

### AACR

Over the 2012 to 2015 period, 5,920 and 2,619 cancer cases were identified in women and men, respectively; 275 were pediatric cancer cases. Of 8,539 total cases, 89% were microscopically verified (cytology/histology examination), and 11% were verified by clinical and other laboratory investigations. None of the cases had an unknown basis of diagnosis. Comparison of incidence rates for the five commonest tumors in women and men is presented in the Data Supplement.

**Fig 1:**
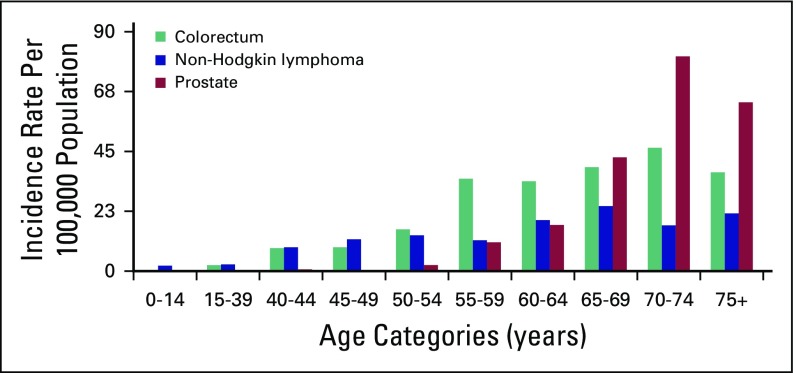
Incidence of the three commonest cancers in men across different age categories.

**Fig 2:**
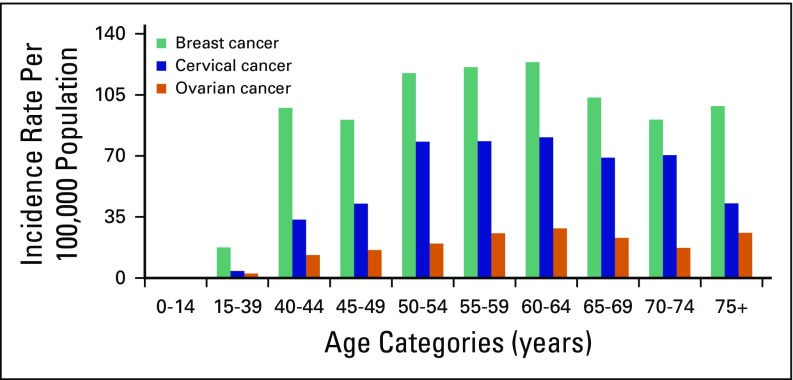
Incidence of the three commonest cancers in women across different age categories.

For 2015, we estimated 21,563 (95% CI, 17,416 to 25,660) and 42,722 (95% CI, 37,412 to 48,040) incident cancer cases in men and women, respectively, with a male-to-female ratio of approximately 1:2. The most common cancer in men age ≥ 15 years was colorectal cancer (CRC), followed by non-Hodgkin lymphoma (NHL), prostate cancer (CaP), leukemia, and lung cancer (Table 1). Disaggregation by age showed an increasing trend in incidence of CRC, NHL, and CaP with age, the highest incidence being older than age 65 years (Fig 1). In females 15 years and older, the most common cancer was breast cancer (BC), followed by cervical cancer (CC), ovarian cancer, CRC, and leukemia (Table 2). BC constituted 33% of the cancers in women and 23% of all cancers in AACR. Disaggregation by age showed increasing incidence of breast and ovarian cancers with age, with the highest incidence observed among 60- to 64-year-olds (Fig 2). For CC, a sharp increase in incidence started with 40- to 44-year-olds and peaked for 60- to 64-year-olds (Fig 2). Over 4 years, among all BC cases identified in Addis Ababa, 12% had complete staging information: most (61%) were stage III and above.

**Table 1:**
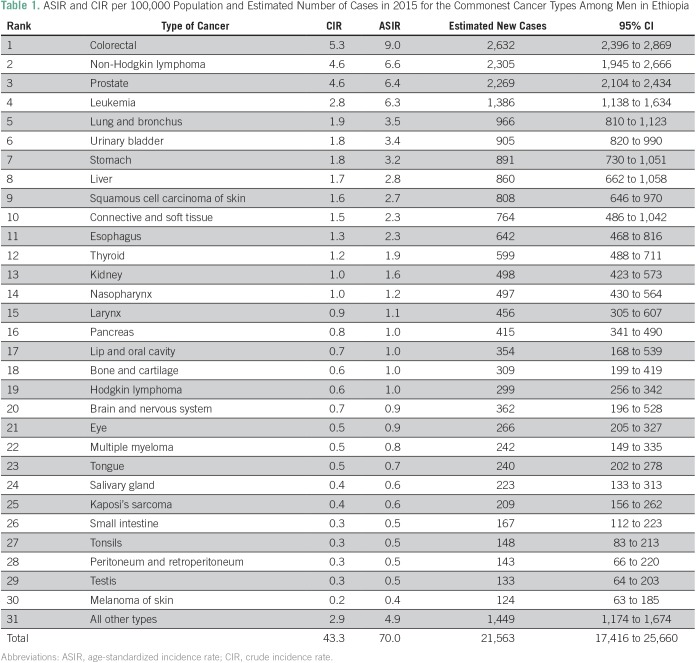
ASIR and CIR per 100,000 Population and Estimated Number of Cases in 2015 for the Commonest Cancer Types Among Men in Ethiopia

**Table 2:**
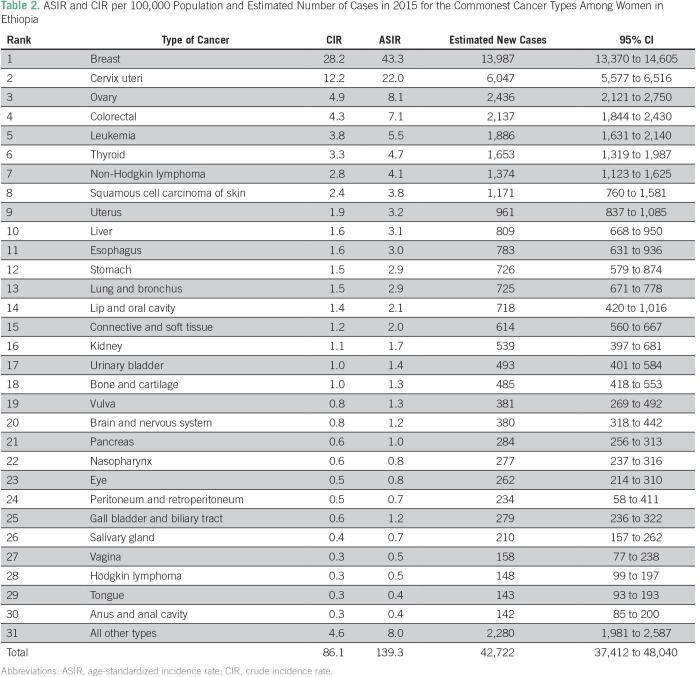
ASIR and CIR per 100,000 Population and Estimated Number of Cases in 2015 for the Commonest Cancer Types Among Women in Ethiopia

We estimated that 3,707 of all cases occurred in the pediatric age group, with leukemia being the commonest cancer (29%), followed by NHL, Wilms tumor, and retinoblastoma (Table 3). Acute leukemia accounted for 89% (of which 91% was acute lymphocytic leukemia and 9% was acute myeloid leukemia) of all the leukemia cases in children.

**Table 3:**
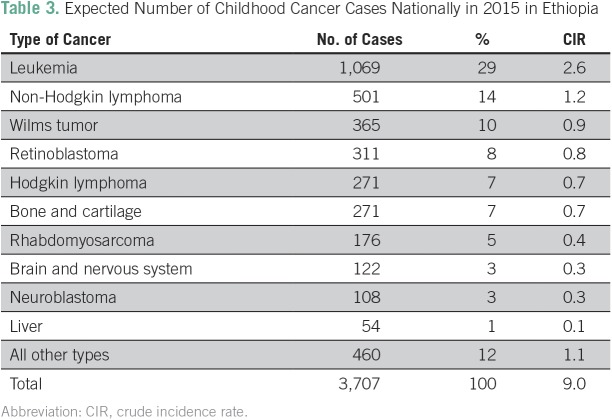
Expected Number of Childhood Cancer Cases Nationally in 2015 in Ethiopia

### Regional Data

A total of 1,648 cancer cases were identified from regional hospital-based pathology units in 2015 (Table 4): 540 cases from Amhara, 653 from Tigray, 139 from SNNP, 244 from Oromia and Harari, and 72 from Dire Dawa. All of the cases identified were microscopically verified: 543 (33%) were among men and 1,105 (67%) among women.

**Table 4:**
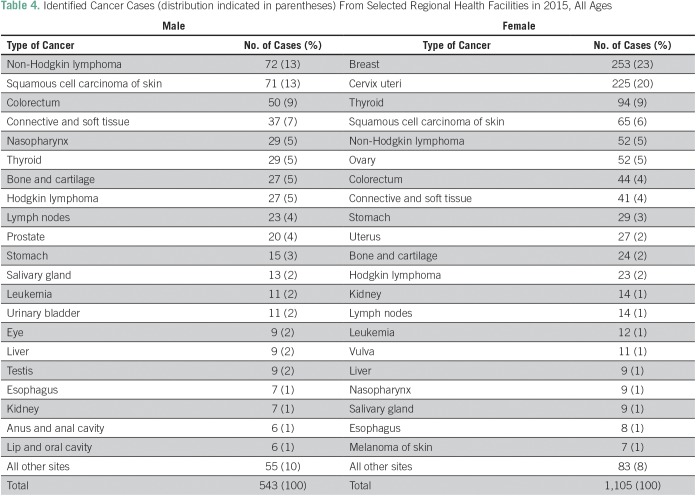
Identified Cancer Cases (distribution indicated in parentheses) From Selected Regional Health Facilities in 2015, All Ages

Regional data showed that NHL was the most common tumor in men, followed by squamous carcinoma of the skin and CRC. Fewer cases of leukemia and CaP were identified in the regions. In women, BC was the commonest malignancy, followed by CC, but with variation across the different regions. In Amhara and Tigray, CC was the leading cause.

## DISCUSSION

The CIR and ASIR presented are 4-year averages (2012 to 2015) from AACR, from which we estimated the national burden of cancer in Ethiopia. Given possible variation in incidence of different types of cancer over the 4 years, such average rates are likely to be better than a single-year estimate. The 10 commonly occurring cancers in Ethiopia were found to be cancers of the breast and cervix, colorectal cancer, NHL, leukemia, and cancers of the prostate, thyroid, lung, stomach, and liver. Most of these cancers have either known preventable risk factors or available screening programs for early detection and treatment that have paramount impact on clinical outcomes.^1^ Even though there are variations in ASIR, the commonest cancers identified are comparable with GLOBOCAN estimates (Data Supplement), but with notable differences.^9^ With GLOBOCAN, Kaposi sarcoma was the third (ASIR of 6.1 cases per 100,000) and eighth (ASIR of 3.1 per 100,000) commonest cancer in men and women, respectively.^9^ On the contrary, Kaposi sarcoma was not among the most common cancers in our estimation (ASIR < 0.5 per 100,000). Kaposi sarcoma is a type of cancer that commonly occurs in patients with advanced HIV infection. Unlike neighboring countries, adult HIV prevalence in Ethiopia is low, approximately 1% (2015), with antiretroviral treatment coverage of 65% creating unfavorable conditions for a large occurrence of Kaposi sarcoma.^21^ Likewise, contrary to GLOBOCAN, we found that connective and soft tissue and bone and cartilage cancers were more common in Ethiopia.

The incidence of all cancers combined was twice as high among women than among men. The disparity is essentially due to the high rates of BC and CC among women. Although cancer incidence is higher among men in more developed nations, higher incidence was also observed in women from Eastern Africa.^22^ There is no clear understanding of what underlies the sex disparity in cancer risk. Differences could be influenced by genetic factors, sex-specific hormones, and differential exposure to risk factors.^23^ Low detection and underreporting of CaP due to absence of screening in Ethiopia could influence the observed sex disparity.

BC was the commonest cancer, constituting 23% of all cancers. Although BC incidence varies among different countries, several reports confirm that it is the most common tumor in women, and its incidence is increasing in Africa.^24,25^ Similar to other African countries, most cases presented at late stage with associated higher mortality levels.^26^ Wide-scale introduction of screening programs, such as clinical breast examination and ultrasound, prompt diagnosis, and high-quality treatment can greatly improve BC outcomes.^27^ Although mammography is an important screening modality for early detection of BC in high-income countries (HICs), wide-scale introduction in LICs is limited, owing to its cost and complex resource requirements.^27^ As for CC, introduction in Ethiopia of visual inspection of the cervix with acetic acid wash and immediate treatment of precancerous lesions with cryotherapy is a promising initiative.^28,29^ Scale-up of screening services and developing capacity to treat identified cases would have an impact on CC incidence and mortality.^29,30^ A comprehensive approach to CC control would include introduction of human papilloma virus vaccine into the national immunization program targeting adolescent girls.^30^

Estimated national BC ASIR (43 per 100,000) is on par with previous estimates for Ethiopia and other Eastern African countries (ASIRs of 34 to 46 per 100,000).^9,24^ However, our CC estimate (22 per 100,000) is slightly lower than GLOBOCAN (26 per 100,000) and substantially different from what has been reported in Uganda (46 per 100,000), which could be partly explained by Uganda’s higher HIV prevalence.^8,9^ Despite our BC and CC estimates being close to those of GLOBOCAN, they could be an overestimation or underestimation. Our estimates were based on data from an urban setting, where BC risk factors (eg, contraceptive usage, lower fertility) and lifestyle factor differences (eg, obesity, early menarche, late menopause) are highly prevalent compared with rural settings where the majority of Ethiopians live.^16,31^ Although BC was most common in the capital, CC was the leading cause of cancer in two of the six regions included in the study. The observed differences identified in the regions may reflect true differences in incidence (which would have implications on national estimates) or variations in health care access and limited capacity in identifying some types of cancer that require imaging modalities. Furthermore, regional data were collected from pathology units only and could not give a comprehensive picture because they did not capture cases identified through clinical or other laboratory investigations.

CRC is the third most common cancer, with ASIR of 8 per 100,000. ASIRs of 7 and 8 per 100,000 were also reported in Zimbabwe and Uganda, respectively.^8^ It was more common in men than women, similar to the pattern observed globally.^1,32^ CRC is declining in HICs like the United States.^33,34^ Identifying and removing precancerous polyps using sigmoidoscopy/colonoscopy screening seems to have contributed substantially to the decline.^33^ Although a generalized screening policy may be feasible in HICs, its application in LICs such as Ethiopia should be weighed in light of severe resource constraints and competing priorities.^34^

Leukemia was most common (nearly 30% of all cancers) in children, and similar findings were reported elsewhere.^35^ Other common childhood cancers included lymphomas, Wilms tumor, retinoblastoma, bone and cartilage cancer, and rhabdomyosarcoma. Elsewhere, brain tumor is one of the commonest malignancies, but it represents only 3% of identified pediatric cancers in Ethiopia, which may relate to the complexity of diagnosing brain tumors in children where imaging studies are limited.^35,36^ Unlike adult cancers, most childhood malignancies are not caused by modifiable risk factors, which limits the effectiveness of public health interventions on their incidence. The prognosis of pediatric cancers in Ethiopia remains poor, but in HICs, advances in treatment have resulted in a 5-year survival rate of approximately 80%.^35,36^ Intensity of chemotherapy that matches disease risk and improvements in supportive care to prevent and manage treatment complications are key aspects of success. Intense chemotherapy regimens decrease cancer relapse rates but could result in treatment-related mortality, more so in LICs, where supportive care capacity is lacking. A better option in Ethiopia would be to use graduated-intensity regimens (less-intensive regimens first and intensify treatment once therapy is found safe and effective).^37^ Success in cancer care is also dependent on stable drug supplies and educational and other interventions aimed at supporting families to decrease care abandonment.^38^

CaP is the most frequently diagnosed cancer among men in HICs (ASIR of 124 per 100,000).^1^ Estimates from sub-Saharan Africa also show that CaP is the leading cause of cancer in men, although incidence is generally lower than in HICs, with ASIRs of 18 per 100,000 (from 5 in Niger to 60 in South Africa).^39^ Our data (ASIR of 6 per 100,000) reveals a lower rate compared with the sub-Saharan African average; possible reasons include limited health care access, lack of screening, and under-reporting. Another possible reason is the population’s age structure and competing causes of mortality, because metastatic disease mainly manifests during older age. These factors are important limitations that may generally lead to underestimation of incidence of most cancers included in our analysis. Furthermore, an important challenge for population-based cancer registries in LICs is their dependence on the level of health care access that may not be universal; some patients use alternative care or die at home before seeking care, which is more prevalent in those residing in rural areas.

We have assessed here cancer incidence in Ethiopia, where cancer has become a staggering public health problem. The variation in the cancer types identified in Addis Ababa and other regions calls for expansion of cancer registry sites, including in rural areas. Improving quality and coverage of cancer registration is important to guide future cancer control programs.^40,41^ Prevention and control of NCDs requires a strong health system that has the capacity to develop knowledge, skills, and effective management. The Ethiopian government endeavor to launch health insurance could serve as an important platform for health system strengthening and improve the delivery of quality care for NCDs, including cancer.^14^ The inclusion of cancer treatment within national health insurance programs in the Americas has improved access to services and survival for cancers amenable to treatment, such as cervical, breast, colon, ovarian, and prostate cancer, calling for a targeted approach in the face of resource constraints.^41^ The fight against cancer requires both increased investment in assessing epidemiology and application of existing cancer control knowledge across all segments of the Ethiopian population.
